# Calreticulin Binds to Fas Ligand and Inhibits Neuronal Cell Apoptosis Induced by Ischemia-Reperfusion Injury

**DOI:** 10.1155/2015/895284

**Published:** 2015-10-25

**Authors:** Beilei Chen, Zhengzheng Wu, Jun Xu, Yun Xu

**Affiliations:** ^1^Department of Neurology, Drum Tower Hospital of Nanjing Medical University, Nanjing 210008, China; ^2^Department of Neurology, Northern Jiangsu Province Hospital, Yangzhou 225009, China

## Abstract

*Background*. Calreticulin (CRT) can bind to Fas ligand (FasL) and inhibit Fas/FasL-mediated apoptosis of Jurkat T cells. However, its effect on neuronal cell apoptosis has not been investigated. *Purpose*. We aimed to evaluate the neuroprotective effect of CRT following ischemia-reperfusion injury (IRI). *Methods*. Mice underwent middle cerebral artery occlusion (MCAO) and SH-SY5Y cells subjected to oxygen glucose deprivation (OGD) were used as models for IRI. The CRT protein level was detected by Western blotting, and mRNA expression of CRT, caspase-3, and caspase-8 was measured by real-time PCR. Immunofluorescence was used to assess the localization of CRT and FasL. The interaction of CRT with FasL was verified by coimmunoprecipitation. SH-SY5Y cell viability was determined by MTT assay, and cell apoptosis was assessed by flow cytometry. The measurement of caspase-8 and caspase-3 activity was carried out using caspase activity assay kits. *Results*. After IRI, CRT was upregulated on the neuron surface and bound to FasL, leading to increased viability of OGD-exposed SH-SY5Y cells and decreased activity of caspase-8 and caspase-3. *Conclusions*. This study for the first time revealed that increased CRT inhibited Fas/FasL-mediated neuronal cell apoptosis during the early stage of ischemic stroke, suggesting it to be a potential protector activated soon after IRI.

## 1. Introduction

Ischemic stroke, with high morbidity and mortality, is the most common type of stroke accounting for more than 80% of all stroke cases. It can affect people of all ages. Previous studies have shown both endoplasmic reticulum (ER) stress and neuronal apoptosis are involved in the progression of ischemic stroke [1, 2].

The ER is an organelle responsible for protein synthesis and folding. ER stress occurs under some pathological conditions, such as ischemic stroke [1]. Calreticulin (CRT) is a conserved 46-kDa Ca^2+^ binding protein expressed virtually in all nucleated cells and is located preferentially in the ER [3]. As a chaperone molecular and a modulator of the Ca^2+^ balance, CRT resides mostly in the lumen of the ER, is upregulated during ER stress, and transferred to outside the cytomembrane [4].

Fas (also known as CD95 or APO-1) is a member of the tumor necrosis factor (TNF) receptor family and promotes apoptosis through a well-established pathway. Activation of Fas can recruit and activate caspase-8, a cysteine protease, which then induces cleavage of other protein substrates, such as caspase-3, contributing to neuronal apoptosis [5, 6]. On the cell surface, CRT has been found to bind to FasL (Fas ligand), the ligand of Fas [7], and inhibit Fas/FasL-mediated apoptosis of Jurkat T cells [4, 8]. Given that the Fas/FasL pathway plays an important role in neuronal apoptosis during ischemic stroke [2], we speculated that if CRT expression is increased in the brain after ischemia-reperfusion injury (IRI), CRT may bind to FasL and interfere with Fas/FasL complexation, thereby protecting neurons from apoptosis and relieving ischemia-related damage.

To date, the effect of CRT on neuronal apoptosis has not been investigated. In this study, we observed upregulated CRT expression after ischemia and interaction between CRT and FasL, which led to reduced neuronal cell death, suggesting a possible novel approach to protecting the brain from IRI injury via based on CRT.

## 2. Materials and Methods

### 2.1. Experiment Animals

Kunming mice (10 per group, 25–30 g, 4 weeks old, male : female = 1 : 1) were obtained from the Experimental Animal Center of Nanjing University Medical School, Nanjing, China. Fifty mice were divided into the following five groups (*n* = 10 for each group): sham-operated group and four groups (3 h-MCAO, 6 h-MCAO, 12 h-MCAO, and 24 h-MCAO) in which middle cerebral artery occlusion (MCAO) was performed and mice were killed at 3, 6, 12, or 24 h after reperfusion, respectively. A random block design was carried out using SAS software. The investigators were masked to the group assignments. The protocols for animal experiments were approved by the Animal Care and Use Committee of Nanjing University, Nanjing, China.

### 2.2. MCAO Model

The MCAO model was generated as previously described by Zhang et al. [9]. Briefly, intraperitoneal injection of ketamine (100 mg/mL) and xylazine (20 mg/kg) mixture (1 : 1) (1 mL/kg) was applied to anesthetize the mice. Mice were then subjected to MCAO using 6/0 monofilament nylon sutures with heat-rounded tips inserted through the internal carotid artery (ICA) into the beginning of the middle cerebral artery (MCA). After 2 h of occlusion of the MCA, the filament was withdrawn for blood reperfusion. Three, six, twelve, or twenty-four hours later, the mice were killed and the brain tissues were collected for real-time polymerase chain reaction (PCR) analysis, Western blotting, and immunofluorescence staining. The sham-operated group was treated using the same procedures as described above except MCAO. The mortality of MCAO among the model mice was approximately 10%.

### 2.3. SY5Y Cell Culture and Oxygen Glucose Deprivation (OGD)

SH-SY5Y cells (a neuroblastoma cell line) were provided by the American Type Culture Collection (ATCC) and cultured in Dulbecco's Modified Eagle's Medium (DMEM) containing 10% heat-inactivated fetal bovine serum (FBS, Gibco) with 2 mM glutamine, 100 U/mL penicillin, and 100 *μ*g/mL streptomycin. Cells were maintained at 37°C in a humidified incubator with 5% CO_2_. The* in vitro* OGD model was established as described previously by Tarr et al. [4]. Briefly, an oxygen-depleted, glucose-free medium was applied to SH-SY5Y cells before incubation in a hypoxic chamber (5% CO_2_/95% N_2_) for 15 min. The chamber was sealed, and incubation at 37°C continued for 8 h. After OGD, the cells were returned to normal medium with the presence of different concentrations of CRT (Abcam, ab91577, UK) and incubated for use in subsequent experiments.

### 2.4. Real-Time PCR

Real-time PCR was performed as described previously [10]. Trizol reagent (Takara, Dalian, China) was used to extract total RNA, and a PrimeScript RT reagent kit (Takara) for Quantitative PCR (ABI 7500, Foster City, CA, USA) was used to reverse-transcribe the total RNA into cDNA with the supplement of a fluorescent dye (SYBR Green I; Takara). The primers were as follows: CRT: forward, 5′-TGA TCC CAC AGA CTC CAA GC-3′, reverse, 5′-TCAGCGTATGCCTCATCGTT-3′; caspase-3: forward, 5′-TGT CAT CTC GCT CTG GTA CG3′, reverse, 5′-TCC CAT AAA TGA CCC CTT CA-3′; caspase-8: forward, 5′-GGC CTC CAT CTA TGA CCT GA-3′; reverse, 5′-TGT GGT TCT GTT GCT CGA AG-3′.

### 2.5. Western Blotting

Western blotting was carried out as described previously [11]. A nuclear and cytoplasmic protein extraction kit (Beyotime Institute of Biotechnology, Shanghai, China) was used to prepare cytoplasmic and nuclear proteins. Equal amounts of sample protein were separated by sodium dodecyl sulfate- (SDS-) polyacrylamide gel electrophoresis (PAGE) before blotting onto polyvinylidene fluoride membranes. Membranes were incubated with anti-CRT antibody (1 : 1000, Abcam, ab22683) at 4°C overnight. The protein was detected using horseradish peroxidase-conjugated anti-mouse or anti-rabbit secondary antibodies and visualized using enhanced chemiluminescence detection reagents (Bioworld, Nanjing, China). The intensity of blots was quantified using ImageJ software (National Institutes of Health, Bethesda, MD, USA).

### 2.6. Coimmunoprecipitation (co-IP)

For co-IP, 500 *μ*L PBS with protease inhibitor (1 : 100, Sigma-Aldrich, St. Louis, MO, USA) was used to dilute 500 *μ*g protein. The solution was preincubated with anti-CRT antibody (12.5 *μ*g/mL, Abcam, ab22683) or anti-Fas antibody (1 *μ*g/mL, Santa Cruz Biotechnology Inc., sc-21730, Santa Cruz, CA, USA) at 4°C overnight on a rotating shaker. Additional rotation for 2 h was continued after addition of 20 *μ*L protein A/G-Sepharose bead slurry (Millipore, Billerica, MA, USA) to the mixture. Ice-cold cell lysis buffer was used to wash the slurry three times. Proteins were eluted with SDS sample buffer and boiled for 5 min. Supernatant was subjected to SDS-PAGE and Western blotting using anti-FasL antibody (1 : 200, Santa Cruz Biotechnology, Inc., sc-834).

### 2.7. Immunofluorescence

At 3 h after reperfusion, the SH-SY5Y cells were fixed in 4% paraformaldehyde for 20 min at room temperature and then seeded on cover slips. Monoclonal rabbit anti-CRT antibody (1 : 500, Bioss, bs-5913R, Beijing, China) and monoclonal mouse anti-FasL antibody (1 : 200, Abcam, ab81196) were applied to the cells at 4°C overnight. Then they were incubated with secondary antibody (1 : 200, Invitrogen, Carlsbad, CA, USA) for 45 min at room temperature. The nuclei of the cells were stained using a DAPI kit (KeyGen Biotech, Nanjing, China). Fluorescent images were taken by immunofluorescent microscopy (Olympus, Tokyo, Japan) and laser scanning confocal microscopy (Olympus).

### 2.8. MTT and Caspase-8 and Caspase-3 Activity Assays

The viability of OGD SH-SY5Y cells was assessed using the conventional MTT assay as previously described [12]. The caspase-8 and caspase-3 activities of OGD-exposed SH-SY5Y cells were assessed using caspase activity assay kits (Beyotime Institute of Biotechnology, China) according to the manufacturer's protocol.

### 2.9. Apoptosis Assay by Flow Cytometry

Apoptosis among OGD-exposed cells was assessed using an Annexin V-FITC apoptosis detection kit (KeyGen Biotech, China). Cells (1 × 10^6^/mL) were rinsed twice with PBS and then resuspended in binding buffer containing 5 *μ*L Annexin V-fluorescein isothiocyanate (FITC) and 5 *μ*L propidium iodide (PI). After incubation at room temperature in the dark for 15 min, the cells were analyzed by flow cytometry (FACS Cantoll, Becton Dickinson and Co., Franklin Lakes, NJ, USA) using Diva software.

### 2.10. Statistical Analysis

All data are expressed as mean ± standard error of the mean (SEM). Differences between two groups were determined by independent samples *t*-tests. All statistical analyses were conducted using Statistical Product and Service Solutions 19.0 software (SPSS, Inc., Chicago, IL, USA). A difference was considered statistically significant at *P* < 0.05.

## 3. Results

### 3.1. Increase in CRT Expression in the Cortexes of MCAO Mice at 3 h after Reperfusion, Earlier Than the Increase in Caspase-8 and Caspase-3 mRNA Expression

**Figure 1:**
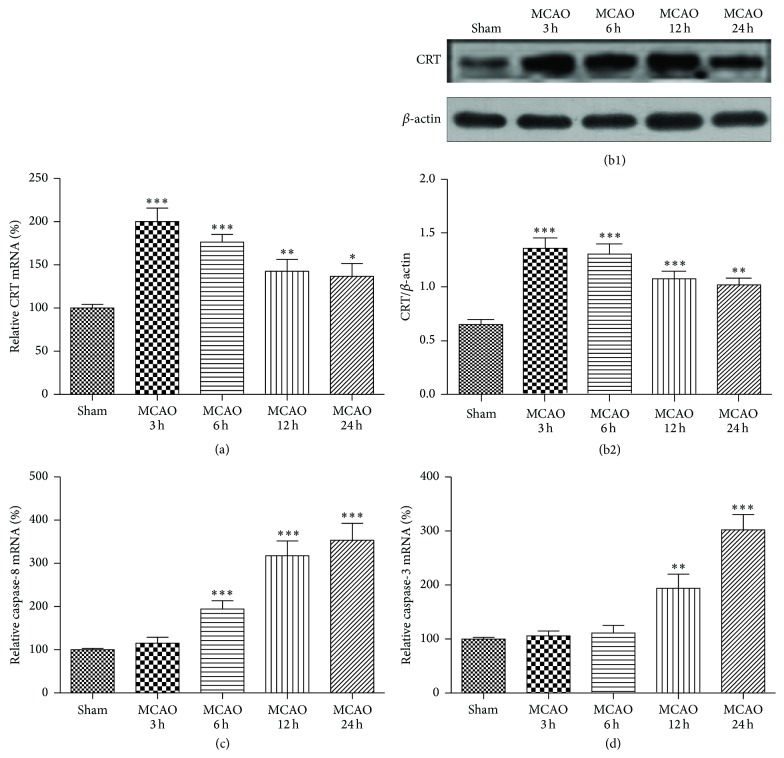
Variation in CRT protein level and mRNA expression of CRT, caspase-8, and caspase-3 in the cortexes of MCAO mice. The ipsilateral cortexes of the sham and MCAO mice were collected at 3, 6, 12, and 24 h after reperfusion. (a) CRT mRNA expression detected by real-time PCR and (b1, 2) CRT protein levels detected by Western blotting. (c) Caspase-8 and (d) caspase-3 mRNA detected by real-time PCR. ^*∗*^ *P* < 0.05, ^*∗∗*^ *P* < 0.01, and ^*∗∗∗*^ *P* < 0.0001 versus sham. *n* = 6 repeats.

To investigate whether and when CRT expression was increased in the cortexes of MCAO mice, real-time PCR and Western blotting were used to detect the mRNA and protein levels of CRT, respectively. Real-time PCR showed that CRT mRNA expression was significantly increased at 3, 6, 12, and 24 h after reperfusion, quickly reaching a peak at 3 h after reperfusion and remaining at high levels for the next 21 hours (200.0 ± 15.63% at 3 h, 176.4 ± 8.88% at 6 h, 142.7 ± 13.66% at 12 h, and 136.8 ± 14.74% at 24 h) (Figure 1(a)). In line with the RT-PCR results, Western blotting showed high CRT levels from 3 h to 24 h after reperfusion with expression at 3 h being the highest (Figures 1(b1) and 1(b2)). We also used RT-PCR to explore when the expression of caspase-8 and caspase-3 was elevated. The increase in caspase-8 mRNA expression became significant 6 h after reperfusion and reached peak levels at 24 h (194.5 ± 18.85% at 6 h, 317.5 ± 34.41% at 12 h, and 353.4 ± 39.29% at 24 h, Figure 1(c)). Similarly, a significant increase in caspase-3 mRNA was detected 12 h and 24 h after reperfusion (193.8 ± 26.42% at 12 h and 302.2 ± 28.18% at 24 h, Figure 1(d)).

### 3.2. CRT Binds to FasL on the Neuronal Cell Surface after Ischemia

**Figure 2:**
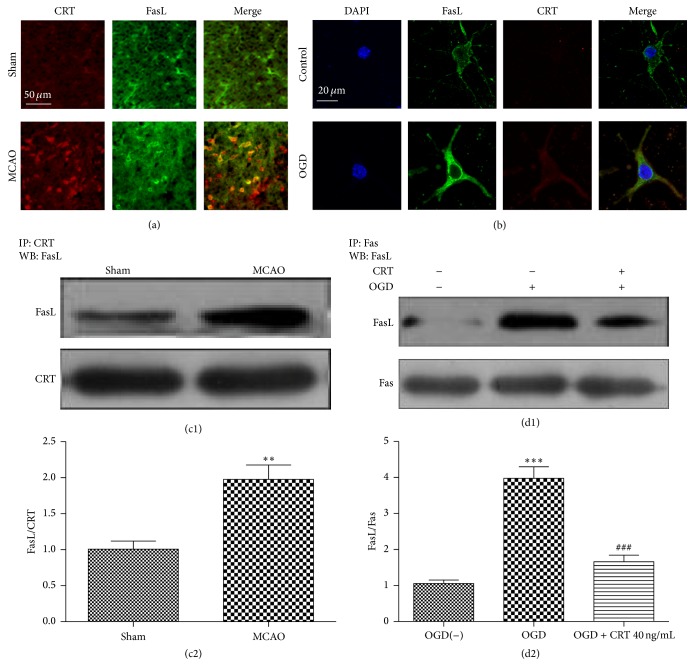
CRT binds to FasL on the surface of neurons after ischemia. (a) Immunofluorescent staining of the cortexes of sham and MCAO mice. (b) Confocal microscopy images of immunostained normal SH-SY5Y cells and OGD-exposed SH-SY5Y cells at 3 h after reperfusion. (c1, 2) Co-IP of CRT and FasL in the cortexes of sham and MCAO mice. (d1, 2) Co-IP of Fas and FasL in normal SH-SY5Y cells and OGD-exposed SH-SY5Y cells cultured with or without 40 ng/mL CRT. ^*∗∗*^ *P* < 0.01, ^*∗∗∗*^ *P* < 0.0001 versus control, and ^###^ *P* < 0.0001 versus OGD group. *n* = 6 repeats.

To examine the expression of CRT and its location in the cortex of ischemic mice, immunofluorescence was used to stain brain slices from sham and MCAO mice. Little CRT was detected in the cortexes of sham mice, whereas a large amount was detected in those mice subjected to MCAO and killed at 3 h after reperfusion. FasL was detected in the brain of both sham and MCAO mice (Figure 2(a)). To further validate whether CRT was expressed on the neuronal cell surface, laser scanning confocal microscope was used to detect the quantity and location of CRT in OGD-exposed SH-SY5Y cells. CRT was barely visible in normal SH-SY5Y cells but became obvious in the cytoplasm and plasma membrane of the OGD-exposed SH-SY5Y cells 3 h after reperfusion and colocalized on the cell surface with FasL (Figure 2(b)). To verify that CRT can bind to FasL, protein was extracted from the cortexes of sham mice and 3 h-MCAO mice. Co-IP was performed, and the results demonstrated that the two proteins can interact with each other in both groups; however, more protein complexes were observed in MCAO mice (Figures 2(c1) and 2(c2)). To confirm that CRT could compete with Fas and prohibit Fas/FasL combination, protein was extracted from SH-SY5Y cells and co-IP was performed. It was found that little Fas formed a complex with FasL in normal SH-SY5Y cells; however, increased interaction was detected in OGD-exposed SH-SY5Y cells cultured without CRT at 3 h after reperfusion. In contrast, with the presence of 40 ng/mL CRT, the amount of Fas/FasL was remarkably lower (Figures 2(d1) and 2(d2)).

### 3.3. CRT Protected OGD-Exposed SH-SY5Y Cells

**Figure 3:**
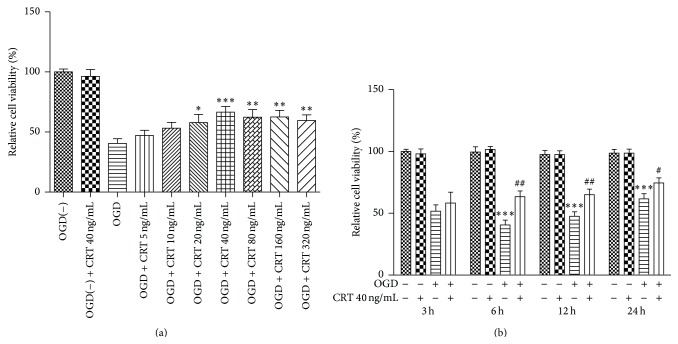
CRT protects SH-SY5Y cells during OGD. SH-SY5Y cells were cultured in media containing different concentrations of CRT after OGD. Cell viability was detected by the MTT assay. (a) Cell viability of SH-SY5Y cells cultured in different concentrations of CRT at 6 h after reperfusion and (b) cell viability of SH-SY5Y cells cultured with or without 40 ng/mL CRT at 3, 6, 12, and 24 h after reperfusion. Control in 3(a): untreated OGD-exposed SH-SY5Y cells. Control in (b): normal SH-SY5Y cells. ^*∗*^ *P* < 0.05, ^*∗∗*^ *P* < 0.01, and ^*∗∗∗*^ *P* < 0.0001 versus control and ^#^ *P* < 0.05, ^##^ *P* < 0.01 versus OGD group. *n* = 6 repeats.

To evaluate the ability of CRT to protect neurons during IRI, the SH-SY5Y cells were moved to medium containing different concentrations of CRT immediately after OGD. The MTT assay was applied to evaluate cell viability at 3, 6, 12, and 24 h after reperfusion. CRT at concentrations of 20–320 ng/mL increased the viability of OGD-exposed SH-SY5Y cells at 6 h after reperfusion from 40% (untreated control) to approximately 60% (Figure 3(a)). Thus, 40 ng/mL was used for subsequent experiments. The protective effects were obvious at 6 h after reperfusion and diminished with time (*P* = 0.0014 at 6 h, *P* = 0.0051 at 12 h, and *P* = 0.0393 at 24 h, Figure 3(b)).

### 3.4. CRT Protects OGD-Exposed SH-SY5Y Cells from Apoptosis and Downregulates the Activity of Caspase-8 and Caspase-3

**Figure 4:**
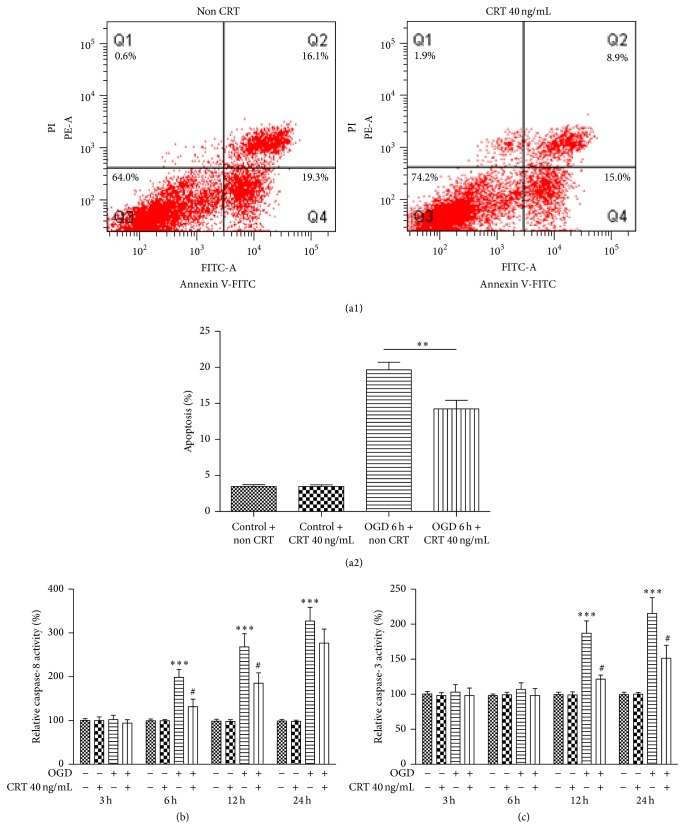
CRT prevents apoptosis among OGD-exposed SH-SY5Y cells and downregulates the activity of caspase-8 and caspase-3. SH-SY5Y cells were cultured in medium with or without 40 ng/mL CRT after OGD. (a1, 2) Apoptosis rate of OGD-exposed SH-SY5Y cells as detected by flow cytometry at 6 h after reperfusion and activity of (b) caspase-8 and (c) caspase-3 as detected using caspase activity assay kits at 3, 6, 12, and 24 h after reperfusion. Control: normal SH-SY5Y cells. ^*∗∗*^ *P* < 0.01, ^*∗∗∗*^ *P* < 0.0001 versus control and ^#^ *P* < 0.05 versus OGD group. *n* = 6 repeats.

To determine whether CRT can protect neurons from apoptosis during ischemia, flow cytometry was used to detect the apoptosis rate and caspase activity assay kits were used to determine the activities of caspase-8 and caspase-3 at 3, 6, 12, and 24 h after reperfusion. The apoptosis rate of control SH-SY5Y cells was similar after 12 h in culture with or without 40 ng/mL CRT (untreated: 3.47 ± 0.26%, CRT treated: 3.46 ± 0.24%, *P* = 0.9778). However, in OGD-exposed SH-SY5Y cells, the apoptosis rate was significantly lower when the cells were treated with 40 ng/mL CRT for 12 h after reperfusion (untreated: 19.66 ± 1.06%, CRT treated: 14.24 ± 1.18%, *P* = 0.0030; Figures 4(a1) and 4(a2)). Compared with cells cultured without CRT, OGD-exposed SH-SY5Y cells cultured in the presence of 40 ng/mL CRT showed significantly less caspase-8 activity at 6 h (untreated: 198.6 ± 18.1%, CRT treated: 131.6 ± 17.2%, *P* = 0.0153) and 12 h after reperfusion (untreated: 268.1 ± 30.1%, CRT treated: 185.1 ± 23.9%, *P* = 0.0443). Similarly, reduced caspase-3 activity was also observed in OGD-exposed SH-SY5Y cells treated with 40 ng/mL CRT at 12 h (untreated: 187.2 ± 17.5%, CRT treated: 121.6 ± 15.7%, *P* = 0.0121) and 24 h after reperfusion (untreated: 215.3 ± 22.5%, CRT treated: 151.4 ± 18.5%, *P* = 0.0418; Figure 4(c)).

## 4. Discussion

This study revealed, for the first time, that CRT is upregulated during the early stage of ischemia and may interact with FasL to inhibit neuronal apoptosis. We first showed that the expression of CRT on the neuron surface was increased after IRI. As an ER chaperone protein, CRT normally resides in the ER and is increasingly expressed during ER stress, which is one of the early pathological processes in ischemic stroke. CRT barely can be detected in normal SH-SY5Y cells. However, higher levels of CRT protein and mRNA were detected in the cortexes of MCAO mice compared with mice in the sham group. As a result of ER stress, highly expressed CRT can be transferred to the plasma membrane [4] and has been demonstrated to be involved in multiple extracellular functions, such as cell attachment, immune thrombosis, and angiogenesis [13, 14].

In this study, we found that one of the major consequences following upregulated CRT expression on the cell surface could be the interruption to the formation of Fas/FasL complex due to competitive binding of CRT with FasL. The binding of CRT to FasL was verified in this study. A previous study showed that, on the T-cell surface, CRT can bind to FasL [7] and inhibit Fas/FasL-mediated apoptosis of T cells in the joints of patients with rheumatoid arthritis [8]. FASL has been reported to be expressed by both neurons and neuroglia [15]. It exists in two forms: a 37-kDa membrane-bound FasL (mFasL) and a 30-kDa soluble FasL (sFasL) [16]. sFasL is a cleaved and soluble form of FasL released from activated cells and is traditionally considered as a cytokine that can induce apoptosis in susceptible cells [16]. We found that, during IRI, CRT was transferred to the neuronal surface and colocalized with FasL. Furthermore, co-IP confirmed that CRT could bind to the 30-kDa sFasL. It is quite possible that upregulated CRT on the neuronal cell surface can bind to sFasL during IRI and, therefore, prevents sFasL from activating the Fas/FasL pathway and triggering neuronal apoptosis.

It has been shown that increased surface expression of CRT is correlated with apoptosis rate. A study by Tarr et al. showed that the spontaneous apoptosis rate of Jurkat cells was about 8.5%, which is the same as the percentage of CRT-positive cells. After treatment with FasL, 24% of the Jurkat cells proceeded to undergo apoptosis, and CRT was expressed on the surface of 44% of these Jurkat cells [4]. Our study showed that, in the cortexes of MCAO mice, CRT mRNA expression was increased obviously at 3 h after reperfusion, which is earlier than the timing of the increase in mRNA expression of downstream signaling molecules of Fas/FasL-mediated apoptosis, that is, caspase-8 and caspase-3. Furthermore, CRT was also visible on the neuronal surface via immunohistofluorescent staining at 3 h after reperfusion, in line with the results of Tarr's study. The earlier increase in CRT expression makes it possible for CRT to inhibit Fas/FasL-mediated apoptosis. Our results showing that the viability of OGD-exposed SH-SY5Y cells was increased by the presence of CRT, most obviously at 6 h and 12 h after OGD, validated that CRT is a potential neuron protector induced soon after IRI. Our study also revealed that the high protein and mRNA expression of CRT faded with time, whereas the mRNA expression of caspase-8 and caspase-3 increased gradually within the first 24 h after reperfusion, suggesting that the protective effect of endogenous CRT may only exist during the early stage of IRI.

A previous study by Duus et al. demonstrated the interaction of CRT and FasL and a subsequent conformational change in FasL [7]. We hypothesize that such a change may prohibit FasL from interacting with its receptor, Fas, and, therefore, decrease the recruitment and activation of its downstream effector proteins. It was observed in our study that the apoptosis rate was lower in OGD-exposed SH-SY5Y cells cultured with the presence of 40 ng/mL CRT than in those cultured without CRT. Our study revealed that, in normal SH-SY5Y cells, the activity of caspase-8, which is first activated by Fas, was increased at 6 h after reperfusion and maintained over the first 24 h. Then the activity of caspase-3, the protein substrate of caspase-8 and inducer of apoptosis, was elevated later than that of caspase-8. We also found that the activities of both caspase-8 and caspase-3 were lower in OGD-exposed SH-SY5Y cells cultured with 40 ng/mL CRT than in those cultured without CRT, further demonstrating the effect of CRT on impeding Fas/FasL-triggered apoptosis. It is worth noting that Fas/FasL is not the only pathway that mediates neuronal apoptosis after ischemic stroke. Many other apoptosis signals, such as TRAIL, TNF-*α*, and Bcl-2 [7, 17, 18], are also involved in neuronal apoptosis following IRI. As a result, CRT only partially inhibited apoptosis in OGD-exposed SH-SY5Y cells in our study.

FasL is involved in many mechanisms of neuronal injury besides apoptosis, such as inflammation and immunity [8, 19]. Thus, the interaction of CRT with FasL may prevent damage to neurons through some other pathways. The ER and non-ER functions of CRT, such as calcium storage and signaling, protein folding, and immunity regulation [13, 20], may also contribute to its effect on neuronal protection.

Our study is limited because SH-SY5Y cells are a neuroblastoma cell line, which differs from normal human neuronal cells in many aspects. For example, the high proliferative capability of the cell line leads to greater cell viability and a lower apoptosis rate after IRI compared to those in primary neurons. Most of our experiments were done* in vitro*, and more complicated* in vivo* experiments are needed to further elucidate the interaction between CRT and FasL. Nevertheless, our data do provide some insights into the neuroprotective effect of CRT in ischemic stroke.

## 5. Conclusions

In conclusion, this study showed that, during the early stage of ischemic stroke, CRT was quickly upregulated within hours, transferred to the surface of neurons, and bound to FasL. This led to inhibition of Fas/FasL-mediated neuronal apoptosis and indicated the potential of CRT in neuron protection following ischemic stroke.

## References

[1] Srinivasan K., Sharma S. S. (2011). Augmentation of endoplasmic reticulum stress in cerebral ischemia/reperfusion injury associated with comorbid type 2 diabetes. *Neurological Research*.

[2] Martin-Villalba A., Herr I., Jeremias I. (1999). CD95 ligand (Fas-L/APO-1L) and tumor necrosis factor-related apoptosis- inducing ligand mediate ischemia-induced apoptosis in neurons. *The Journal of Neuroscience*.

[3] Gold L. I., Eggleton P., Sweetwyne M. T. (2010). Calreticulin: non-endoplasmic reticulum functions in physiology and disease. *FASEB Journal*.

[4] Tarr J. M., Young P. J., Morse R. (2010). A mechanism of release of calreticulin from cells during apoptosis. *Journal of Molecular Biology*.

[5] Jia J., Guan D., Zhu W. (2009). Estrogen inhibits Fas-mediated apoptosis in experimental stroke. *Experimental Neurology*.

[6] Rosell A., Cuadrado E., Alvarez-Sabín J. (2008). Caspase-3 is related to infarct growth after human ischemic stroke. *Neuroscience Letters*.

[7] Duus K., Pagh R. T., Holmskov U., Højrup P., Skov S., Houen G. (2007). Interaction of calreticulin with CD40 ligand, TRAIL and Fas ligand. *Scandinavian Journal of Immunology*.

[8] Tarr J. M., Winyard P. G., Ryan B. (2010). Extracellular calreticulin is present in the joints of patients with rheumatoid arthritis and inhibits FasL (CD95L)-mediated apoptosis of T cells. *Arthritis and Rheumatism*.

[9] Zhang M., Li Q., Chen L. (2014). PSD-93 deletion inhibits Fyn-mediated phosphorylation of NR2B and protects against focal cerebral ischemia. *Neurobiology of Disease*.

[10] Zhao H., Wang S.-L., Qian L. (2013). Diammonium glycyrrhizinate attenuates A*β*(1-42)-induced neuroinflammation and regulates MAPK and NF-*κ*B pathways in vitro and in vivo. *CNS Neuroscience and Therapeutics*.

[11] Li J., Zhang S., Lu M. (2013). Hydroxysafflor yellow A suppresses inflammatory responses of BV2 microglia after oxygen-glucose deprivation. *Neuroscience Letters*.

[12] Zhang Z., Wu Z., Zhu X., Hui X., Pan J., Xu Y. (2014). Hydroxy-safflor yellow A inhibits neuroinflammation mediated by A*β*1-42 in BV-2 cells. *Neuroscience Letters*.

[13] Gold L. I., Eggleton P., Sweetwyne M. T. (2010). Calreticulin: non-endoplasmic reticulum functions in physiology and disease. *The FASEB Journal*.

[14] Mans S., Banz Y., Mueller B. U., Pabst T. (2012). The angiogenesis inhibitor vasostatin is regulated by neutrophil elastase-dependent cleavage of calreticulin in AML patients. *Blood*.

[15] Shin D. H., Lee E., Kim H. J. (2002). Fas ligand mRNA expression in the mouse central nervous system. *Journal of Neuroimmunology*.

[16] Imanishi T., Han D. K., Hofstra L. (2002). Apoptosis of vascular smooth muscle cells is induced by Fas ligand derived from monocytes/macrophage. *Atherosclerosis*.

[17] Wang W., Wang T., Feng W.-Y., Wang Z.-Y., Cheng M.-S., Wang Y.-J. (2014). Ecdysterone protects gerbil brain from temporal global cerebral ischemia/reperfusion injury via preventing neuron apoptosis and deactivating astrocytes and microglia cells. *Neuroscience Research*.

[18] Li M., Peng J., Wang M.-D., Song Y.-L., Mei Y.-W., Fang Y. (2014). Passive movement improves the learning and memory function of rats with cerebral infarction by inhibiting neuron cell apoptosis. *Molecular Neurobiology*.

[19] Niu F.-N., Zhang X., Hu X.-M. (2012). Targeted mutation of Fas ligand gene attenuates brain inflammation in experimental stroke. *Brain, Behavior, and Immunity*.

[20] Michalak M., Groenendyk J., Szabo E., Gold L. I., Opas M. (2009). Calreticulin, a multi-process calcium-buffering chaperone of the endoplasmic reticulum. *Biochemical Journal*.

